# Editorial: Holistic prevention strategies for tail biting in pigs; from farm to slaughterhouse

**DOI:** 10.3389/fvets.2023.1296461

**Published:** 2023-11-09

**Authors:** Richard B. D'Eath, Keelin O'Driscoll, Emma Fàbrega

**Affiliations:** ^1^Scotland's Rural College, Edinburgh, United Kingdom; ^2^Teagasc, Fermoy, Ireland; ^3^Institute of Agrifood Research and Technology (IRTA), Monells, Spain

**Keywords:** tail biting behavior, tail docking, precision livestock farming (PLF), swine production, animal welfare

Tail biting, as well as ear and flank biting, remain persistent production, health, and welfare concerns in the pig industry globally. These unwanted biting behaviors are difficult to solve due to their multifactorial etiology, requiring holistic solutions (1–3). Despite a ban on its routine use in the EU, tail docking to reduce tail injury remains widespread. A common thread of the studies included in this Research Topic is finding a way to house and manage pigs to prevent tail biting without tail docking.

The challenge is made more complicated by the existence of at least two main types of tail biting- (i) Two-stage, where re-directed foraging/exploratory behavior escalates from “tail in mouth” to damaging biting, and (ii) Sudden forceful which seems to occur as a novel aggressive tactic through frustration of access to resources such as feed (4). High levels of persistent biting shown by some individual pigs has been labeled as a distinct third type by some authors (obsessive) (4) with a fourth type “epidemic” being identified by Valros (3). Two of the studies here involve either identified which type of tail biting is operating (Bagaria et al.) or changed a risk factor for one form of biting (D'Alessio et al.).

D'Alessio et al. tested the effect of feeder space on ear, tail and flank injuries and behavior. Twelve groups of undocked pigs had a double feeder space allocation compared to 12 with a single feeder allocation. Although competition and aggression at the feeder was reduced, tail biting was not reduced in the groups with more feeder space. Seven tail biting outbreaks occurred in the 24 groups, with 31% of pigs experiencing some loss of tail length, despite fresh grass in racks, and either rubber floor toys or wooden planks being provided. Thus, although the risk for sudden forceful biting in relation to feeder access was reduced, this did not translate to reducing the risk of tail biting in the rest of the pen.

Taking a different approach, Bagaria et al. studied tail-related and other behaviors in tail-docked weaned piglets under 9 weeks of age, analyzing their inter-relationships using Principle Components Analysis to better understand the context and likely cause of tail biting. Even at this young age early-stage tail biting which caused minor damage was seen: 10% of piglets had minor scratches, although no biting severe enough to cause a wound occurred. Pigs who performed tail biting behaviors the most also performed non-harmful explorative behavior, suggesting that these pigs' biting followed an etiology corresponding to the “2-stage” model of tail biting (4). Such detailed individual-level studies will help researchers to identify the type(s) of tail biting they are dealing with.

Another major challenge of tail biting is that it can occur in unpredictable ways. It can occur in some groups and not others under the same conditions, at varying severity, and it shows unpredictability in time, with apparently sudden “outbreaks” occurring, which then escalate to affect many more pigs. The ability to automatically monitor, and particularly to spot early stages of tail biting before it becomes too severe, would be very valuable, and various “precision livestock farming” approaches, using technology to monitor pigs have been tried.

To this end, Hakansson and Jensen present a new machine vision approach to tail biting detection, based on features of the entire pen of undocked pigs. Past approaches include D'Eath et al. (5, 6) which relied on detection of (low) tail posture, Liu et al. (7) which first tracked pigs, then used Convolutional Neural Networks (CNNs) for feature detection. Here CNNs for feature detection (pigs biting tails- tail in mouth) in single images were combined with long short-term memory (LSTM) networks or a further CNN to detect short sequences of behavior that characterize tail biting across multiple images. The models were also tested on unseen video images of different groups, and the CNN-LSTM approach was found to generalize better. Pre-weaned piglets (sows were present in the pen) were used- which highlights how early tail biting can begin. Indeed, the effect of pre-weaning risk factors on the development of tail-biting were reviewed recently (8).

Using another PLF approach, Larsen and Pedersen present a detailed study of group drinking patterns in growing/finishing pigs, characterizing the typical diurnal patterns in drinker visits and water consumption, and identifying effects of stocking density on frequency and location of drinker use. Presence/absence of straw, and tail docking had no effect on drinker use, but a decrease in stocking density increased both water use and activation frequency, suggesting that pigs at the standard space allowance could have had restricted access to the drinkers; future work could investigate whether increasing drinker allowance could reduce biting associated with access to resources, similar to D'Alessio et al.'s findings in relation to feeder access. Some pens had tail biting events, defined as one or more pigs with a bleeding wound identified during thrice-weekly tail scoring, and these pens had higher water use and drinker visit frequency. However, the timing of this was not predictably linked to tail biting in order to make it a useable early warning sign. The authors suggest that using RFID at the drinker to record drinking patterns at the individual pig level might yield better results. Automated drinker flow recorders are cheaper and simpler than machine vision cameras, so if this could be made to work reliably it could be attractive to farmers.

Overall, the studies included in this Research Topic provide valuable insight into some of the risks for tail biting, as well as potential methods that can be used to predict and reduce its occurrence. Despite the multifactorial root causes of tail biting, after decades of research, much is now known about how to reduce it, and to manage pigs with intact tails, for example by following the example of Switzerland (9), Sweden (10), or Finland (11). In the EU (and UK) the problem remains a lack of enforcement of the existing ban on routine tail docking and requirement to provide manipulable materials, and regulations which still allow high stocking densities. In addition, the continued use of systems with fully slatted floors make it challenging to provide sufficient loose manipulable materials. Tail docking remains a cheap “solution” to the problem of tail biting and economic incentives to produce long-tailed pigs may be needed (12). Methods to standardize and perhaps automate abattoir scoring of undocked (13) and uninjured (14) tails would support such economic incentives.

## Author contributions

RD'E: Writing—original draft, Writing—review & editing. KO'D: Writing—original draft, Writing—review & editing. EF: Writing—original draft, Writing—review & editing.
